# Supplementary material for: The In Silico Characterization of Monocotyledonous α-l-Arabinofuranosidases on the Example of Maize
Source: Life (Basel). 2023 Jan 18;13(2):266. doi: 10.3390/life13020266 (PMC9964162; doi:10.3390/life13020266)
Supplement: Supplementary file 1 [file life-13-00266-s001.zip › Supplementary_Material_1 revI_Alsu.pdf]

The *in silico* Characterization of Monocotyledonous α-L-Arabinofuranosidases on the Example of Maize

Alsu Nazipova*[1], Olga Makshakova[2], Liudmila Kozlova [1]

[1] Kazan Institute of Biochemistry and Biophysics, FRC Kazan Scientific Center of RAS, the Laboratory of Plant Cell Growth Mechanisms, 420111, Lobachevsky Str. 2/31, Kazan, Russia

[2] Kazan Institute of Biochemistry and Biophysics, FRC Kazan Scientific Center of RAS, the Laboratory of Biophysical Chemistry of Nanosystems, 420111, Lobachevsky Str. 2/31, Kazan, Russia

**Corresponding author, e-mail: nazipova_alsu@mail.ru Orchid-ID: 0000-0002-7746-8985*

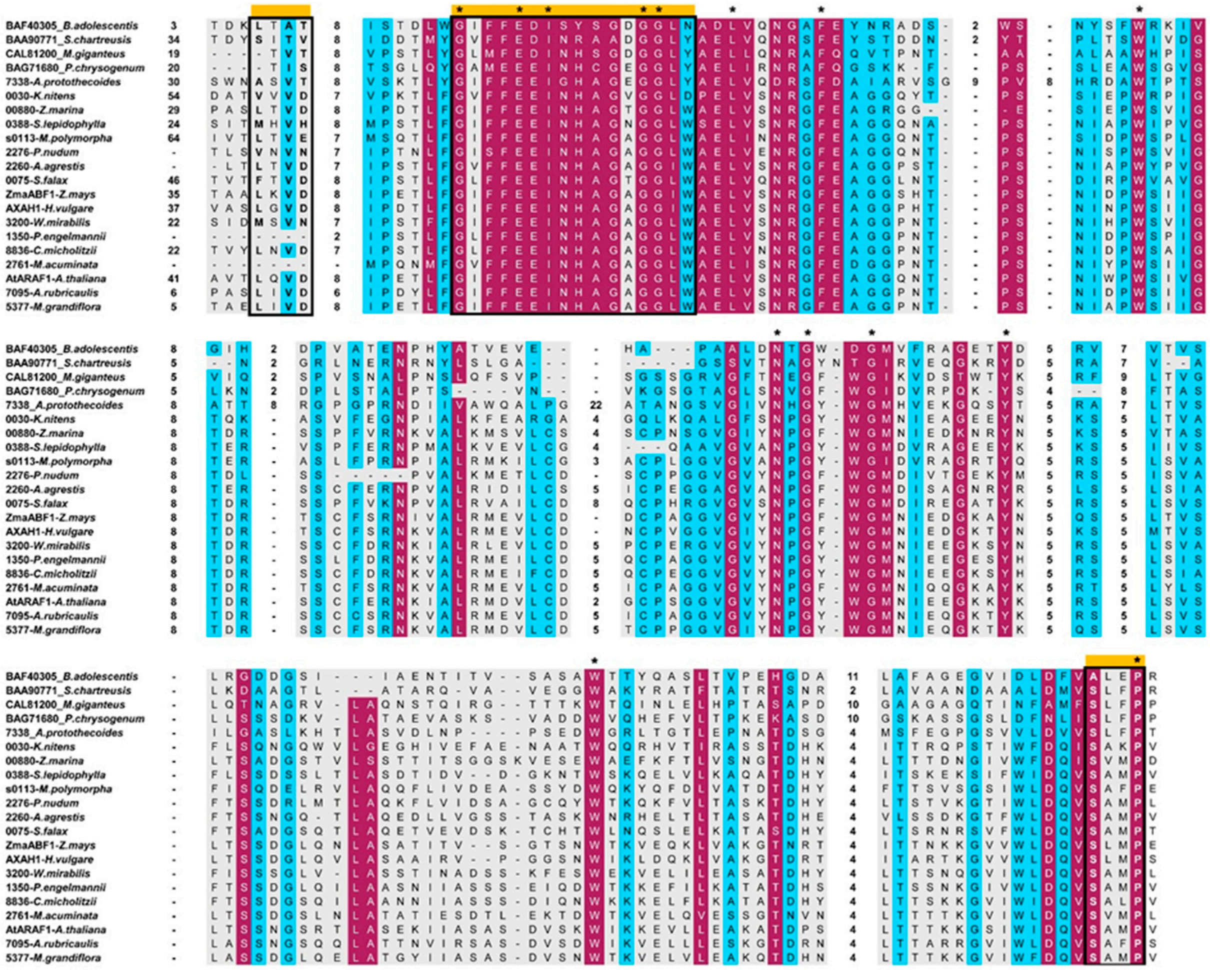

Figure S1. Multiple sequence alignment of CBM4-like domains of GH51 proteins of different origin. The different color of the amino acid background in the multiple alignment indicates the percentage of conservation position. Purple, blue, and gray backgrounds indicate amino acids with 70-100%, 40-69%, and 0-39% conservation, respectively. The asterisks indicate the 100% conserved amino acids in presented alignment. Numbers indicate the number of omitted amino acids. Black rectangles with a yellow stripe on top indicate conservative motifs, which from left to right show the beginning of the CBM4-like domain, the motif which form the bottom of the catalytic site, and the end of the CBM4-like domain, respectively.

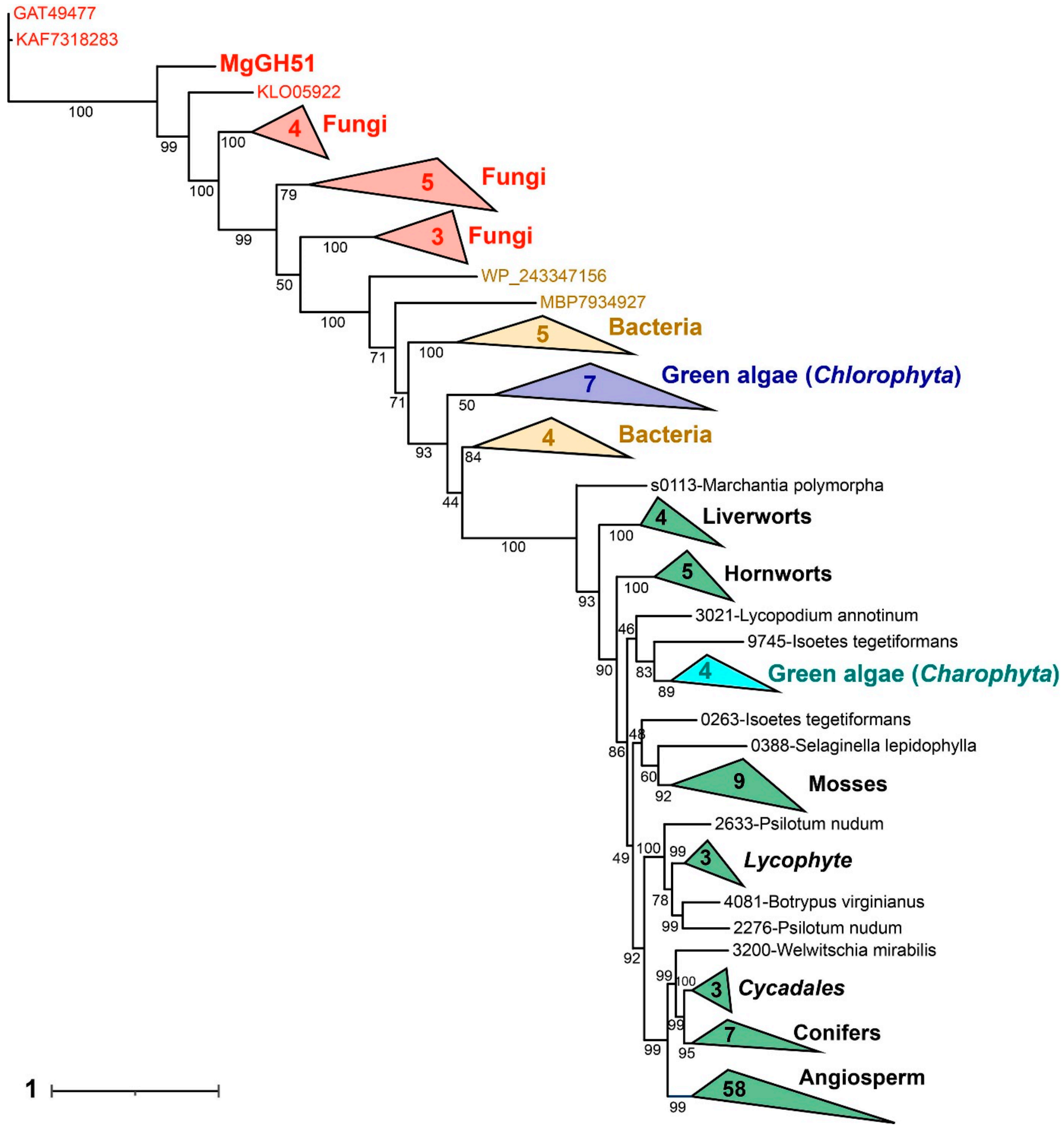

Figure S2. Maximum likelihood phylogenetic tree of the N-terminal CBM4-like domain sequences of fungal, bacterial and plant origin. Clades are collapsed. The green triangles show the collapsed clades of land plants. The names of fungal proteins are red, bacteria are yellow, *Chlorophyta* algae are dark blue, *Charophyta* algae are blue, and the names of land plant proteins are black. The numbers in bold indicate the number of sequences in the collapsed clades. Tree is unrooted, however fungal sequence shown as a root. The numbers indicate the ultrafast bootstrap support values. The full names and sequences of proteins used to build tree are listed in Table S1 in Table S2.

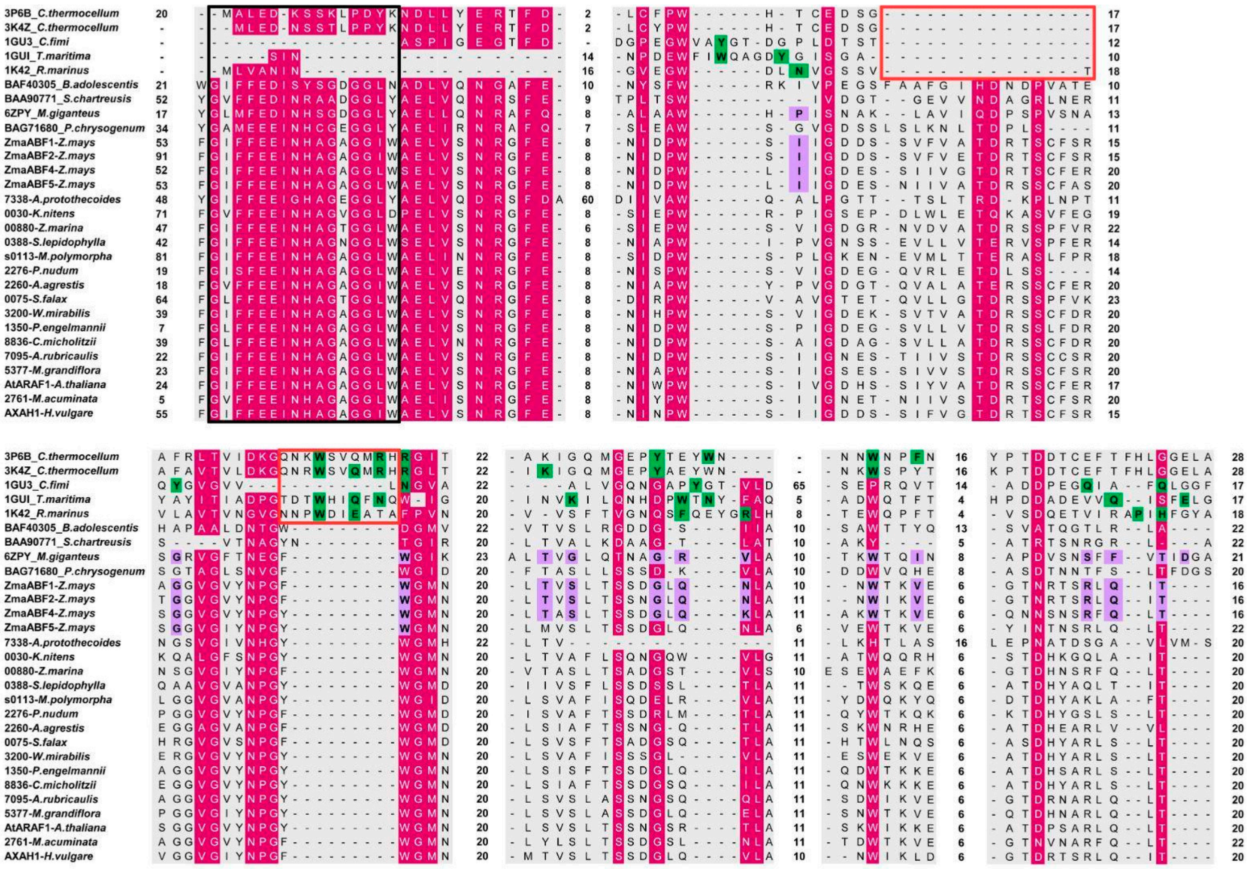

Figure S3. Multiple sequence alignment of CBM4-like domains of bacterial, fungal and plant origin with those bacterial CBM4 domain sequences which crystal structures were resolved. The different color of the amino acid background indicates the percentage of conservation position. Amino acids with 70-100% conservation are crimson and ones with 0-69% are gray. Numbers indicate the omitted amino acids. The black frame shows the conserved motif of the CBM4-like domain in GH51 enzymes. The red frame indicates insertions and deletions in protein sequences of bacterial CBMs of family 4. The amino acid that are involved in substrate recognition and accommodation in CBM4 of *Clostridium thermocellum* cellulase K (PDB ID: 3P6B), CBM4 of *C. thermocellum* cellobiohydrolase A (3K4Z), CBM4-1 of *Cellulomonas fimi* cellulase 9B (1GU3), CBM4-2 of *Thermotoga maritima* laminarinase 16A (1GUI), CBM4-2 of *Rhodothermus marinus* xylanase (1K42) (Boraston et al. 2002; Simpson et al. 2002; Alahuhta et al. 2010, 2011) are highlighted in green. The homologous residues in α-L-arabinofuranosidase of MgGH51 (*Meripilus giganteus*; 6ZPY) (McGregor et al. 2020) and maize GH51 proteins are given in purple.

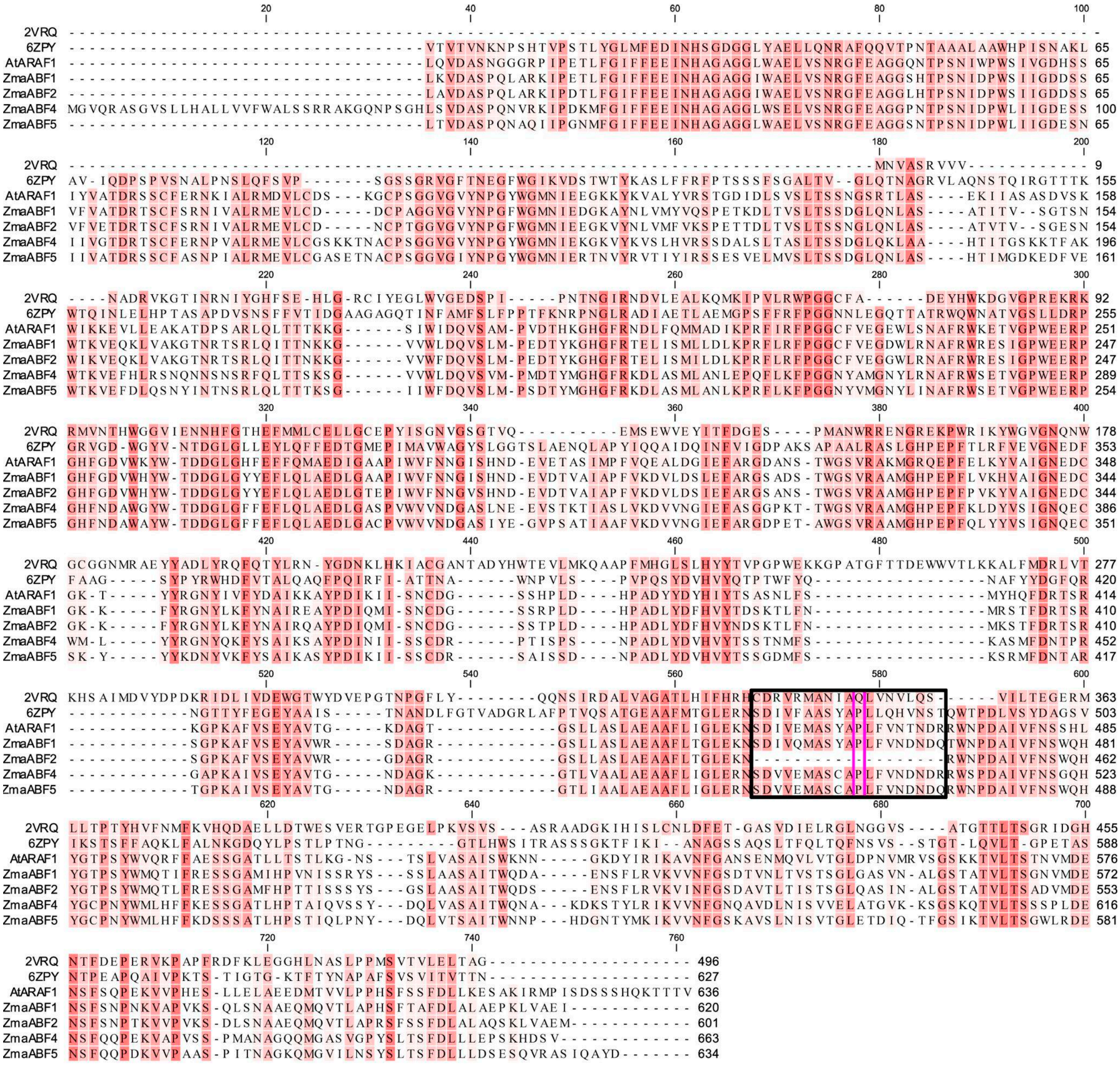

Figure S4. Multiple sequence alignment of maize GH51 proteins and AtARAF1 of *Arabidopsis thaliana* with the sequences of Tx-Abf (PDB ID: 2vrq) from *Thermobacillus xylanilyticus* and MgGH51 (6zpy) from *Meripillus giganteus*. The protein sequences of structural models of ZmaABF1, ZmaABF2, ZmaABF5, and AtARAF1 are shown. Amino acid residues are colored in a white-red gradient corresponding to the percentage of conservation (from 50 to 100%). The black frame indicates the 19-amino acid deletion in ZmaABF2. Residues determining the size of the arabinose-binding pocket in the spatial structures of GH51 proteins are shown by magenta frame.

References

Alahuhta M, Luo Y, Ding S-Y, Himmel ME, Lunin V V (2011) Structure of CBM4 from Clostridium thermocellum cellulase K. Acta Crystallogr Sect F Struct Biol Cryst Commun 67: 527–530

Alahuhta M, Xu Q, Bomble YJ, Brunecky R, Adney WS, Ding S-Y, Himmel ME, Lunin V V (2010) The unique binding mode of cellulosomal CBM4 from Clostridium thermocellum cellobiohydrolase A. J Mol Biol 402: 374–387

Benson DA, Cavanaugh M, Clark K, Karsch-Mizrachi I, Lipman DJ, Ostell J, Sayers EW (2017) GenBank. Nucleic Acids Res 45: D37

Berman HM, Westbrook J, Feng Z, Gilliland G, Bhat TN, Weissig H, Shindyalov IN, Bourne PE (2000) The Protein Data Bank. Nucleic Acids Res 28: 235–242

Boraston AB, Nurizzo D, Notenboom V, Ducros V, Rose DR, Kilburn DG, Davies GJ (2002) Differential oligosaccharide recognition by evolutionarily-related β-1, 4 and β-1, 3 glucan-binding modules. J Mol Biol 319: 1143–1156

McGregor NGS, Turkenburg JP, Mørkeberg Krogh KBR, Nielsen JE, Artola M, Stubbs KA, Overkleeft HS, Davies GJ (2020) Structure of a GH51 α-l-arabinofuranosidase from Meripilus giganteus: conserved substrate recognition from bacteria to fungi. Acta Crystallogr Sect D Struct Biol 76: 1124–1133

Schrödinger, LLC (2015) The {PyMOL} Molecular Graphics System, Version~1.8

Sela I, Ashkenazy H, Katoh K, Pupko T (2015) GUIDANCE2: accurate detection of unreliable alignment regions accounting for the uncertainty of multiple parameters. Nucleic Acids Res 43: W7–W14

Sievers F, Wilm A, Dineen D, Gibson TJ, Karplus K, Li W, Lopez R, McWilliam H, Remmert M, Söding J (2011) Fast, scalable generation of high-quality protein multiple sequence alignments using Clustal Omega. Mol Syst Biol 7: 539

Simpson PJ, Jamieson SJ, Abou-Hachem M, Karlsson EN, Gilbert HJ, Holst O, Williamson MP (2002) The solution structure of the CBM4-2 carbohydrate binding module from a thermostable Rhodothermus marinus xylanase. Biochemistry 41: 5712–5719
